# Deep artificial neural network based on environmental sound data for the generation of a children activity classification model

**DOI:** 10.7717/peerj-cs.308

**Published:** 2020-11-09

**Authors:** Antonio García-Domínguez, Carlos E. Galvan-Tejada, Laura A. Zanella-Calzada, Hamurabi Gamboa, Jorge I. Galván-Tejada, José María Celaya Padilla, Huizilopoztli Luna-García, Jose G. Arceo-Olague, Rafael Magallanes-Quintanar

**Affiliations:** 1 Unidad Académica de Ingeniería Eléctrica, Universidad Autónoma de Zacatecas, Zacatecas, Zacatecas, México; 2 LORIA, Université de Lorraine, Nancy, France; 3 CONACYT, Universidad Autónoma de Zacatecas, Zacatecas, Zacatecas, México

**Keywords:** Children activity recognition, Environmental sound, Machine learning, Deep artificial neural network, Environmental intelligence, Human activity recognition

## Abstract

Children activity recognition (CAR) is a subject for which numerous works have been developed in recent years, most of them focused on monitoring and safety. Commonly, these works use as data source different types of sensors that can interfere with the natural behavior of children, since these sensors are embedded in their clothes. This article proposes the use of environmental sound data for the creation of a children activity classification model, through the development of a deep artificial neural network (ANN). Initially, the ANN architecture is proposed, specifying its parameters and defining the necessary values for the creation of the classification model. The ANN is trained and tested in two ways: using a 70–30 approach (70% of the data for training and 30% for testing) and with a k-fold cross-validation approach. According to the results obtained in the two validation processes (70–30 splitting and k-fold cross validation), the ANN with the proposed architecture achieves an accuracy of 94.51% and 94.19%, respectively, which allows to conclude that the developed model using the ANN and its proposed architecture achieves significant accuracy in the children activity classification by analyzing environmental sound.

## Introduction

Environmental intelligence is an area of artificial intelligence that has made great progress in recent years (Cook, Augusto & Jakkula, 2009), mainly driven by the development of new devices and sensors that facilitate data capture and processing (Stipanicev, Bodrozic & Stula, 2007). Advances in this area have gone hand in hand with the development of the Internet of Things (IoT) (Wortmann & Flüchter, 2015; Li, Da Xu & Zhao, 2015) and Smart Cities (Albino, Berardi & Dangelico, 2015; Arasteh et al., 2016), in which can be found different intelligent systems created to provide services to population. The most recent developments have focused on applications that facilitate the interaction of human beings with their environment, in different areas such as engineering (Gupta et al., 2014; Corno & De Russis, 2016), medicine (Roda et al., 2017; Ziuziański, Furmankiewicz & Sołtysik-Piorunkiewicz, 2014), energy (Robinson, Sanders & Mazharsolook, 2015; Cristani, Karafili & Tomazzoli, 2015), ambient assisted living (Lloret et al., 2015; Blasco et al., 2014; Memon et al., 2014), among others (Al Nuaimi et al., 2015; Hu & Ni, 2017). Many of the projects developed and implemented in this area rely on human activity recognition (HAR) systems, which base their operation on the use of different sensors as a data source to determine the activity that a person or group of people are performing and with this information provide some kind of service (Alahi et al., 2016; Uddin, 2019; Cippitelli et al., 2016; Burbano & Carrera, 2015; Ignatov, 2018; Rafferty et al., 2017).

In works related to human activity recognition and classification, different data sources have been used to collect information about the activity to be analyzed. The most common data sources used are video (Caba Heilbron et al., 2015; Boufama, Habashi & Ahmad, 2017), audio (Liang & Thomaz, 2019; Galván-Tejada et al., 2016), Radio Frequency Identification (RFID) devices (Li et al., 2016; Wang & Zhou, 2015) and smartphones sensors (Wang et al., 2016), such as the accelerometer (Lee, Yoon & Cho, 2017). Another important aspect to consider is the group of people to whom the study is directed, since the type of data source to use, the techniques and algorithms used depend on it. Most applications on human activity recognition and classification are designed considering adults as the group of interest (Reyes-Ortiz et al., 2016; Hammerla, Halloran & Plötz, 2016; Concone, Re & Morana, 2019; Brophy et al., 2020; Lyu et al., 2017). Another group of people for which works are commonly developed are the elderly, especially for automatic assisted living and health care (Capela, Lemaire & Baddour, 2015; Sebestyen, Stoica & Hangan, 2016; De la Concepción et al., 2017). Children are a group for which it is also common to develop applications for monitoring and safety (Mannini et al., 2017; Kurashima & Suzuki, 2015; Trost et al., 2018).

The children’s activities recognition and classification is a topic that has attracted the attention of many researchers due to the implications and variables involved. There are several factors to consider such as: (1) Number of individuals. The number of children acting simultaneously is undoubtedly an important aspect to be considered, since the complete design of the system changes if activities for 1, 2, 3 or a group of children are analyzed. (2) Age of children. Because the activities that children perform are related to their age, the set of activities considered for the analysis changes for each defined group of children. (3) Place of analysis of activities. The environment in which the activity analysis is performed is also an important factor to be considered, since there are external factors that can affect some processes (e.g., a noisy environment would affect the data capture process when working on human activity recognition using environmental sound). And (4) Data source. The type of data used for the analysis of activities is a fundamental variable for the system, since its entire design depends on this. If you work with images, audio, video, embedded sensors or audio, the way to act changes in each situation.

In the area of children activity recognition and classification common data sources are video cameras, accelerometers and RFID devices (Westeyn et al., 2011; Boughorbel et al., 2010; Shoaib et al., 2015; Mannini et al., 2017). Most of the works presented in this area perform the data capture process using wearable sensors, making it possible to identify 2 types of disadvantages:

1. Interference with the children’s natural behavior. Wearing wearable sensors can cause the children, by their curious instincts, to become distracted by feeling a foreign object to him and not perform activities normally (e.g., crying because they want to remove the sensor). This type of behavior would cause an alteration in the captured data or that these were not representative of the activities commonly performed by the children and that are being subject to analysis.
2. Possible physical damage to sensors. The fact that the sensor is located in one of the clothes or parts of the children’s body makes possible the risk that the sensor may suffer physical damage while the children perform the activities, because they are unpredictable (e.g., to wet or hit a smart watch). The sensors are generally not made for rough use, so improper use or even physical damage could represent inappropriate data capture for the system.

To mitigate the problem of using wearable sensors, it is necessary to use a different data source that does not interfere with the analyzed activities, for this purpose sound has been used in some works as data source (Galván-Tejada et al., 2016; Sim, Lee & Kwon, 2015). Capturing audio data has the advantage that the capture device does not necessarily have to be carried by the individual who is performing the activity, but can be located in a nearby place where it does not interfere with the performed activities. In addition, it is not necessary to use special equipment or sensors to capture data, since it is possible to use the microphone present in smartphones.

Machine learning is a branch of artificial intelligence that, since its appearance, has been applied to a wide variety of problems due to the good results it has shown in terms of data analysis, especially in classification problems, such as the presented in this work. Machine learning has the particularity of being able to be applied to an endless number of problems, with different types of data to analyze. Different applications based on machine learning for the analysis of audio data have been developed, among the main commercial applications developed are voice assistants, such as Alexa (Hoy, 2018; Kepuska & Bohouta, 2018), Siri (Aron, 2011; Kepuska & Bohouta, 2018) and Cortana (Bhat, Lone & Paul, 2017; Hoy, 2018), for the Amazon, Apple and Microsoft companies respectively, as well as Google’s voice assistant (López, Quesada & Guerrero, 2017). In machine learning, specifically deep neural networks have also been widely used for analysis of audio data, as in WaveNet (Van der Oord et al., 2016), a deep neural network for generating raw audio waveforms. It is also common to find works based on machine learning focused on audio data classification (Zhang et al., 2019; Zeng et al., 2019; Lu, Zhang & Nayak, 2020; Piczak, 2015; Rong, 2016; Hershey et al., 2017).

For the generation of a human activity recognition and classification model, it is necessary to implement a machine learning classification algorithm (Jordan & Mitchell, 2015; Nikam, 2015; Fatima & Pasha, 2017), which after being trained with a set of training data, is able to classify new data among the analyzed classes. Some of the most commonly used classification algorithms in this area are Support Vector Machine (SVM) (Chen et al., 2017), k-Nearest Neighbor (knn) (Paul & George, 2015), Random Forests (RF) (Uddin, Billah & Hossain, 2016), Extra Trees (ET) (Uddin & Uddiny, 2015) and Artificial Neural Networks (ANN) (Ronao & Cho, 2016; Suto & Oniga, 2018; Murad & Pyun, 2017). In recent years there have been numerous works based on ANN focused on the creation of activity recognition models, due to the performance and efficiency they have shown (Ronao & Cho, 2016; Hassan et al., 2018; Jiang & Yin, 2015; Nweke et al., 2018; Lubina & Rudzki, 2015; Myo, Wettayaprasit & Aiyarak, 2019).

The accuracy of the classifier model depends on the algorithm used and the analyzed data. When working with audio, it is possible to extract features of the signals to serve as input for the classification algorithms. The number and type of features extracted depends on the application and the type of analysis to be performed. Previously, we presented a work that implemented the classification algorithms SVM, kNN, Random Forests, Extra Trees and Gradient Boosting in the generation of a children activity classification model using environmental sound data, working with a 34-feature set extracted from the audios samples, and the classifier that achieves a higher accuracy was kNN with 100% (Blanco-Murillo et al., 2018). In addition, a work was previously presented where the same classification algorithms mentioned above were analyzed and a 27-feature subset was used for the generation of the models, achieving accuracies greater than 90% (Garca-Domnguez et al., 2019). Continuing the previous works, we also developed a children activity recognition and classification model using environmental sound through a 5-feature subset, chosen by genetic algorithms. In that work, the same classifying algorithms mentioned in the previous works were used and the maximum accuracy obteined was 92% (Garca-Dominguez et al., 2020).

In the present work, the architecture of a deep ANN is proposed for its implementation as machine learning algorithm in the generation of a children activity recognition model using environmental sound, in an environmental noise-free environment and analyzing activities of children acting individually. A 34-feature set is extracted from the analyzed dataset, which is used in the generation of the model. The classification model is trained and evaluated in terms of accuracy to obtain the performance of the classification algorithm. Two validation approaches are used: 70–30 split (70% of the data for training and 30% for testing), and a k-fold cross validation.

This document is organized as follows: the materials and methods are described in detail in “Materials and Methods”. In “Experiments and Results” the experiments performed and the results obtained are reported. The discussion and conclusions are described in “Conclusions”. Finally, future work is presented in “Future Work”.

## Materials and Methods

This section describes in detail the used dataset, made up of audio recordings of the different activities to be analyzed, as well as the extracted features used to generate the classification model. Likewise, the methodology used throughout the experimentation is described. For the design and implementation of the deep ANN presented in this work, the Python programming language is used (Van Rossum, 2007), through the Keras (Chollet, 2018) library, an API (Application Programming Interface) of high-level ANN, and the Tensorflow Abadi et al. (2016) library, which is the most widely used deep learning platform today.

### Dataset description

The data set used for this work is the same as the used in previous works about children activity recognition (Garca-Domnguez et al., 2019, 2020) and no extra processing was done. The audios analyzed belong to four different activities, shown in Table 1.

**Table 1:** General description of activities.

Activity	Description
Crying	Emitting crying sound in reaction to some event
Playing	Handling plastic pieces
Running	Moving quickly from one place to another
Walking	Moving from one place to another at medium speed

As shown in Delgado-Contreras et al. (2014), Tarzia et al. (2011), 10-s audio clips seem to be long enough to obtain potentially useful information in the process of classifying activities by analyzing environmental sound. In the dataset used, the audio recordings with a duration greater than 10 s were divided to generate a greater number of samples.

For the analysis of the activities, 10-s samples are taken. Each of the audio recordings in the dataset used belongs to a single activity, so the produced 10-s clips also belong to a single activity. Table 2 shows the number of audio recordings and 10-s samples that the dataset has for each activity to be analyzed.

**Table 2:** Recordings and audio clips per activity.

Activity	Generated		Taken from Internet		Total	
	Recordings	Clips	Recordings	Clips	Recordings	Clips
Crying	8	72	33	532	41	604
Playing	9	67	17	636	26	703
Running	9	81	30	611	39	692
Walking	10	65	30	652	40	717

As shown in Table 2, the dataset used for the presented work consists of 2,716 10-s audio clips and the amount of audios per class is balanced (22.23%, 25.88%, 25.47% and 26.39% crying, playing, running and walking, respectively). There is no rule to define the minimum size of a dataset analyzed using machine learning techniques, the only general recommendation is that it be as large as possible, but that is in particular for each case and type of generated model (e.g., Barbedo (2018) analyzed the size of the dataset for plant disease classification, Dutta & Gros (2018) did it for the classification of medical images and Oyedare (Oyedare & Park, 2019) did it for the classification of transmitters through deep learning). And precisely one of the points to perform as future work for the present work is an analysis of the dataset and consider expanding it if necessary.

For the feature extraction process, a set of numerical features are extracted from the 10-s intervals of the audio signals, these features are shown in Table 3.

**Table 3:** Extracted features.

Feature ID	Feature name
1	Zero crossing rate
2	Energy
3	Entropy of energy
4	Spectral centriod
5	Spectral spread
6	Spectral entropy
7	Spectral flux
8	Spectral rollof
9–21	MFCCs
22–33	Chroma vector
34	Chroma deviation

These 34 extracted features are those commonly used in audio analysis and activity recognition through audio (Scheirer, 1998; Wang et al., 2003), especially the mel-frequency spectral coefficients, since many audio analysis works that make use of them have been developed (Galván-Tejada et al., 2016; Stork et al., 2012; Mascia et al., 2015). This set of features was extracted using Python programming language (Van Rossum, 2007).

### Artificial neural networks

**Figure 1:**
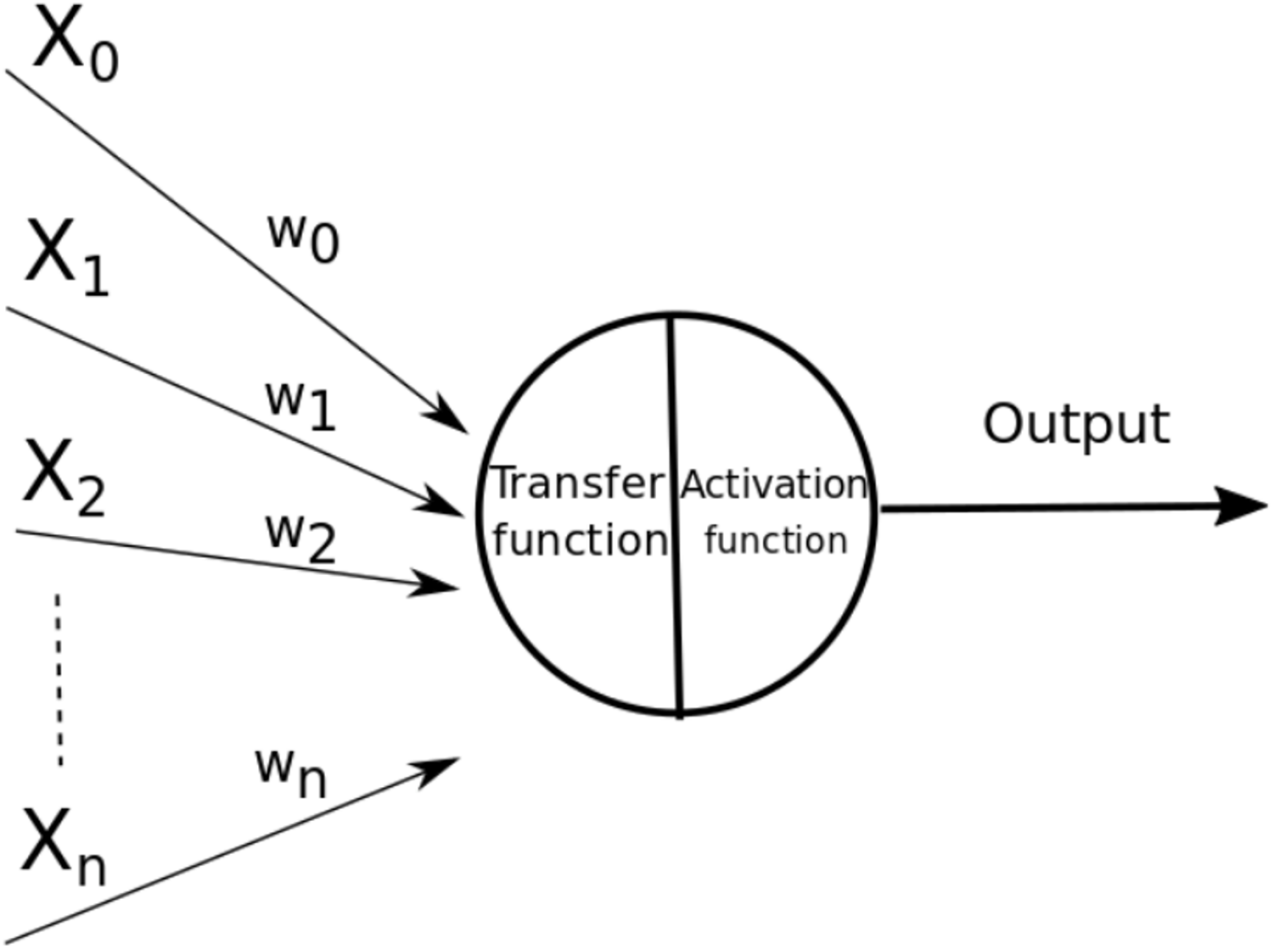
Parts of an artificial neuron (*X_i_* represents the inputs, *w_i_* represents the weights).

An ANN is a model based on the neural structure of the brain, which basically learns from experience. This type of models learn to perform tasks using sample data, without the need to be programed with a specific task. An ANN is composed by nodes called neurons connected to each other. Each of these connections, called edges, are channels that can transmit information from one neuron to another (Monteiro et al., 2017). These edges, regularly, are associated with a real number, called weight, which increases or decreases the signal that passes through the edges and affects the input to the next neuron. The way to calculate the output of the neurons is by some non-linear function of the sum of their inputs. In Fig. 1 the parts of an artificial neuron are shown, the inputs are represented by *X*_*i*_, and the weights associated with each edge by *w*_*i*_, also within the neuron the transfer and activation functions with which the output is calculated are represented.

**Figure 2:**
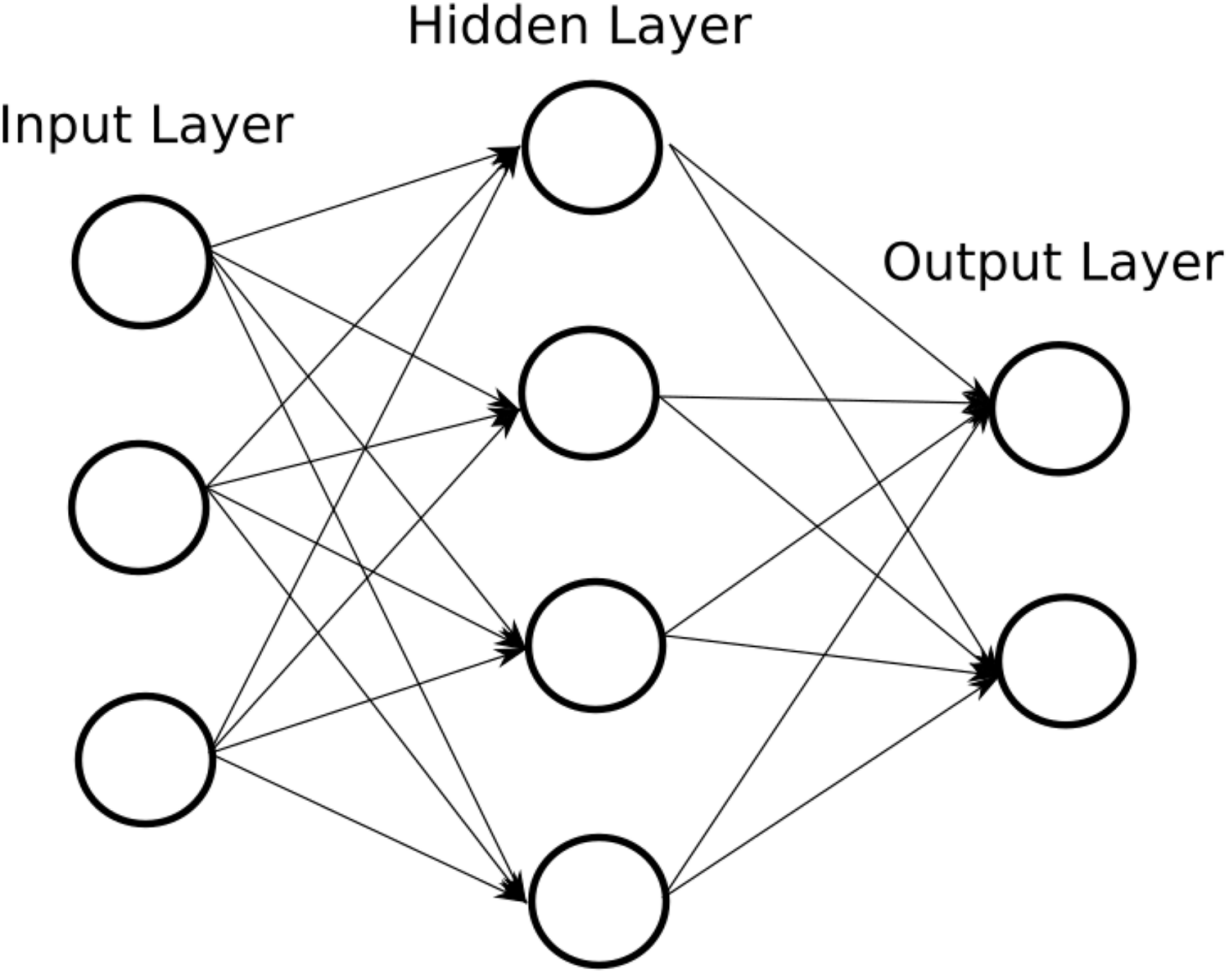
Example of an ANN.

ANN are made up of layers, which can have different types of transformations in their inputs. There are two fully identified layers: the input layer and the output layer (Kia et al., 2012). Therefore, the data travels from the input layer to the output layer, going through the different intermediate layers, called hidden layers. Figure 2 shows an example of a simple ANN.

The number of nodes in the input layer is determined by the number of input data (Monteiro et al., 2017). The data is processed in the hidden layers and the output layer. There is no fixed or recommended number for hidden layers or for their number of nodes, they are regularly tested by trial and error, depending on the application for which the ANN is being designed. When the number of hidden layers is large, as well as the number of nodes in them, it is said that there is a deep ANN. In the same way, the number of nodes in the output layer is determined by the problem to which the ANN applies, in multiclass problems the number of nodes in the output layer corresponds to the number of classes to predict.

### Deep artificial neural network architecture

The ANN architecture definition refers to the structure of the number of layers and nodes contained. There are not strict rules to define the number of hidden layers and the number of nodes, that depends on the problem in which the ANN is implemented and this data is determined by trial and error. One way to select the parameters of ANN, such as the number of nodes and hidden layers, has been to adjust them manually or relying on very deep networks (Simonyan & Zisserman, 2014) that have proved effective in other applications, with the disadvantage of the cost in memory that it implies that the ANN is not optimized for the particular problem and some of these parameters can be redundant (Alvarez & Salzmann, 2016).

### Neural network model

An ANN model is composed by four mainly concepts described in detail below.

#### Type of model

When using the Keras interface for the implementation of an ANN in Python, it is necessary to specify the type of model to be created. There are two ways to define Keras models: Sequential and Functional (Ketkar, 2017). A sequential model refers to the fact that the output of each layer is taken as the input of the next layer, and it is the type of model developed in this work.

#### Activation function

The activation function is responsible for returning an output from an input value, usually the set of output values in a given range such as (0, 1) or (−1, 1). The types of activation functions most commonly used in ANN are:

- Sigmoid: This function transforms the values entered to a scale (0, 1), where high values tend asymptotically to 1 and very low values tend asymptotically to 0 (Karlik & Olgac, 2011), as shown in Eq. (1).
  (1) $$f(x) = \displaystyle{1 \over {1 - {e^{ - x}}}}$$
- ReLU-Rectified Linear Unit: This function transforms the entered values by canceling the negative values and leaving the positive ones as they enter (Yarotsky, 2017), as shown in Eq. (2).
  (2) $$f(x) = \max(0,x) =\bigg\{ {\matrix{0 &  {\rm for} & {{x \lt 0}} \cr x & {\rm for} & {{x \ge 0}}\cr } } \bigg\}$$
- Softmax: This function transforms the outputs into a representation in the form of probabilities, such that the sum of all the probabilities of the outputs is 1. It is used to normalize multiclass type (Liu et al., 2016), as shown in Eq. (3).
  (3) $$f{(z)_j} = \displaystyle{{{e^{{z_j}}}} \over {\sum\nolimits_{k = 1}^k {e^{{z_k}}}}}$$

#### Optimization algorithm

The goal of optimization algorithms is to minimize (or maximize) an objective *E*(*x*) function that is simply a mathematical function that depends on the internal learning parameters of the model that are used to calculate the objective (*Y*) values of the set of predictors (*X*) used in the model. The most commonly used optimization algorithms in ANN are Gradient Descent and Adam (Ruder, 2016; Kingma & Ba, 2014).

#### Loss function

The loss function, also known as the cost function, is the function that indicates how good the ANN is. A high result indicates that the ANN has poor performance and a low result indicates that the ANN is performing positively. This is the function that is optimized or minimized when back propagation is performed. There are several mathematical functions that can be used, the choice of one depends on the problem that is being solved. Some of these functions are:

- Cross-Entropy: Cross-entropy loss, or log loss, measures the performance of a classification model whose output, *y*, is a probability value, *p*, between 0 and 1, and it is calculated with Eq. (4). Cross-entropy loss increases as the predicted probability diverges from the actual label. This function is used for classification problems (Rubinstein & Kroese, 2013).
  (4) $$- (y\,\log(p) + (1 - y)\log(1 - p))$$
- Categorical Cross-Entropy: Also called Softmax Loss (Koidl, 2013). It is a Softmax activation plus a Cross-Entropy loss and it is calculated with Eq. (5), where the double sum is over the observations *i*, whose number is *N*, and the categories *c*, whose number is *C*. The term 1_*y_i_*__∈__*C_c_*_ is the indicator function of the *i*th observation belonging to the *c*th category. The *p*_model_[*y*_*i*_ ∈ *C*_*c*_] is the probability predicted by the model for the *i*th observation to belong to the *c*th category. When there are more than two categories, the ANN outputs a vector of *C* probabilities, each giving the probability that the network input should be classified as belonging to the respective category. When the number of categories is just two, the neural network outputs a single probability ${\hat y_i}$, with the other one being 1 minus the output. This is why the binary cross entropy looks a bit different from categorical cross entropy, despite being a special case of it.
  (5) $$- \displaystyle{1 \over N}\sum\limits_{i = 1}^N \sum\limits_{c = 1}^C {1_{{y_i} \in {C_c}}}\log\,{p_{\rm model}}\left[ {{y_i} \in {C_c}} \right]$$
- Mean Squared Error: Mean Square Error (MSE) is the most commonly used regression loss function (Christoffersen & Jacobs, 2004). MSE is the sum of squared distances between our target variable and predicted values, and it is calculated with Eq. (6), where *n* represents the number of samples and ${\hat y_i}$ represents the predicted value.
  (6) $${\rm MSE} = \displaystyle{{\sum\limits_{i = 1}^n {{({y_i} - {{\hat y}_i})}^2}} \over n}$$

## Experiments and Results

The dataset used in this work is contained by 2,672 10-s audio samples, with 34 extracted features from each sample. Two validation approaches were used: 70–30 split and a k-fold cross validation. For the 70–30 split approach, a training subset and a testing subset are randomly selected, contained by 70% and 30% of the data, respectively (The approach of 70% for training and 30% for testing is used in this area as valid, as proposed by Balli, Sağbaș & Peker (2019), Taylor et al. (2020) and Sousa Lima et al. (2019)). Table 4 shows the number of audio samples in each subset for this case.

**Table 4:** Size of training and test data sets for the 70–30 validation approach.

Total samples	Training data samples	Test data samples
2,672	1,870	802

For the k-fold cross-validation, *k* = 10 was selected, since it is an approach used in works on human activity recognition to estimate an average accuracy and evaluate the model performance, as in the works presented by Altun & Barshan (2010), Dehghani, Glatard & Shihab (2019), and Jordao et al. (2018). Table 5 shows the number of audio samples in the training and test subsets using the 10-fold cross-validation approach.

**Table 5:** Size of training and test data sets for the 10-fold cross-validation approach.

Total samples	Training data samples	Test data samples
2,672	2,404	268

The ANN architecture is the most important aspect to define, since it directly impacts the accuracy achieved by the network. As mentioned earlier, there are no rules to choose the parameters of the architecture since these are conditioned to the type of application that is given to the ANN and they are determined by trial and error. Table 6 shows the proposed architecture of the deep ANN used in this work. The selected parameters for the ANN architecture, as well as the characteristics of the dataset (classes balanced in quantity, proportion of the size of the testing and training subsets, number of features), ensure that no overfitting appears for the generated model, based on the obtained results, so no dropout layers were used.

**Table 6:** Proposed ANN architecture.

Inputs	Hidden layers	Neurons per layer	Outputs	Batch size	Epochs
34	8	20	4	64	200

In Table 7 the selected parameters for the development and implementation of the model in the Keras interface with Python are presented. For the choice of the used parameters in the model implementation, those that best adapt to the type of problem in which they are being applied (multiclass classification) were taken and some of them are present by default in the keras interface, considering that they are generally the ones that achieve the best performing (e.g., the ReLU activation function is the most successful and widely used activation function (Ramachandran, Zoph & Le, 2017)).

**Table 7:** Selected model parameters.

Type of model	Sequential
Input layer activation function	Relu
Hidden layers activation function	Relu
Output layer activation function	Softmax
Loss function	Categorical crossentropy
Optimization algorithm	Adam

**Figure 3:**
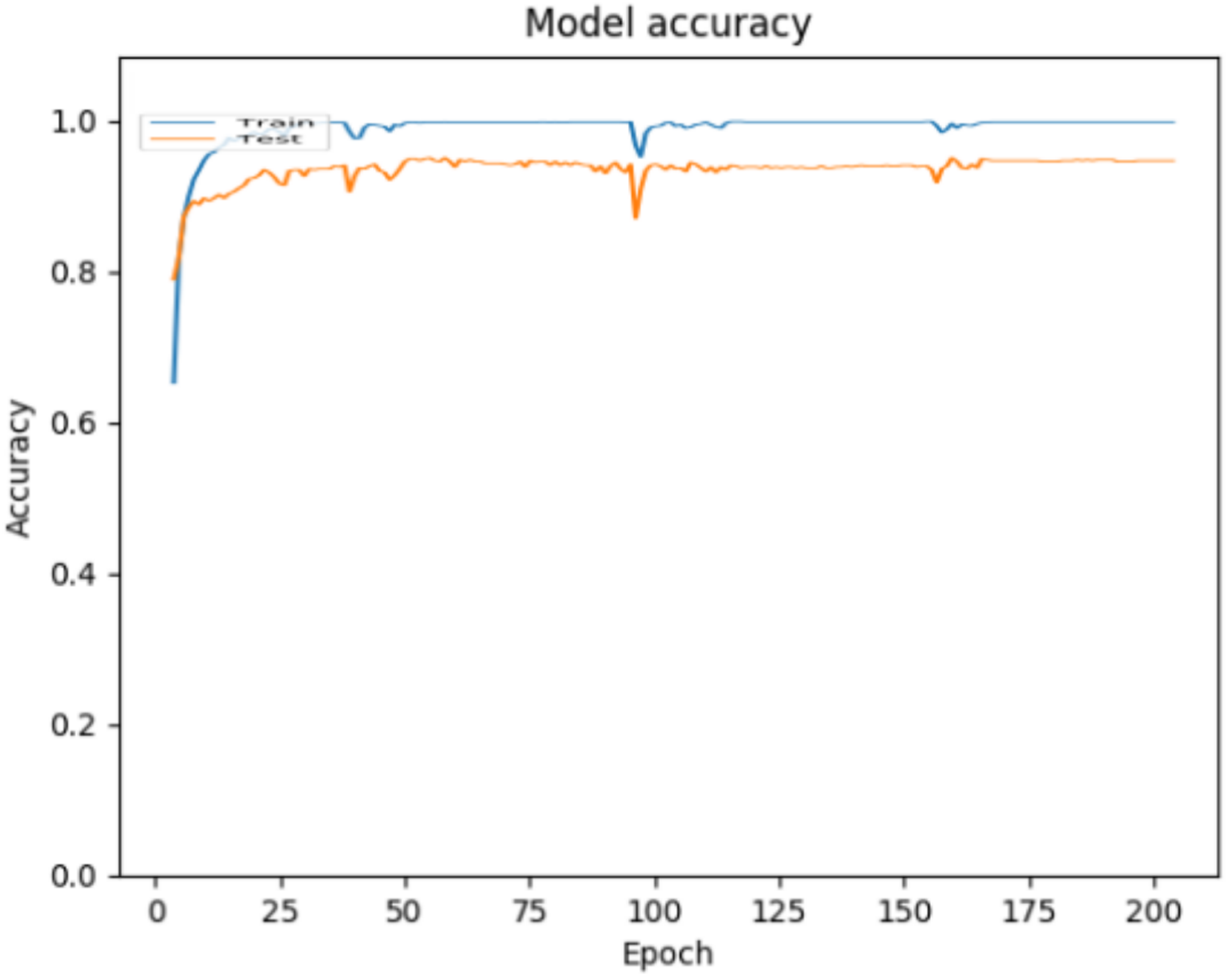
Accuracy behavior during ANN training and testing for the 70–30 split approach.

The ANN model created with the described architecture and parameters was implemented, executed and validated in the Python programming language. As mentioned above, two validation approaches were used to evaluate the performance of the model. For the 70–30 split approach, Fig. 3 shows the accuracy achieved by the model over epochs, where can be observed that the accuracy achieved for the classification of children’s activities using environmental sound is 0.9979 for training data and 0.9451 for testing data.

**Figure 4:**
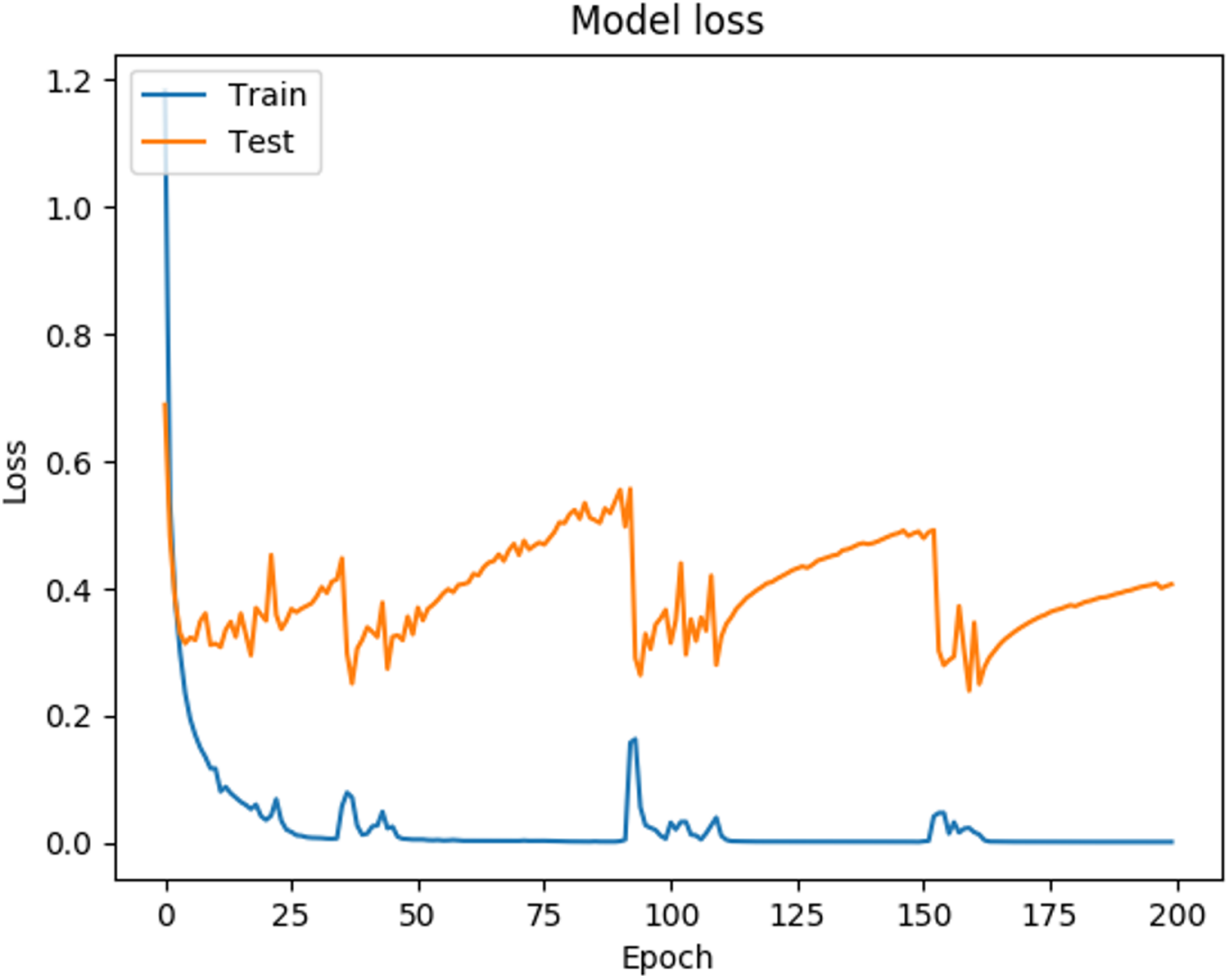
Loss function behavior during ANN training and validation.

In Fig. 3 it can be observed that the curves representing the model accuracy (training and testing accuracy) periodically spikes down, which is a consequence of the training process being stuck in local regions, due to the optimization parameters used (The Adam optimization function was used, with the default parameters (Keras Team, 2020). Figure 4 shows the loss presented by the ANN, where can be observed that the loss for the training data is 0.0018, while for the testing data is 0.4070, which is related to the general evaluation of the ANN and indicates how good it is for the classification.

In Fig. 4 it can be observed that the curves representing the model loss (training and testing loss) present irregular spikes, which is due properly to the shape of the analyzed data and the parameters chosen in the network architecture, specifically the Adam optimization function, as mentioned above.

From the results obtained by executing the classification model with the 70–30 split approach, it is also possible to analyze the behavior of the model specifically for each of the activities analyzed in this work. Table 8 shows the confusion matrix generated from the execution of the classification model for the testing data. In the confusion matrix it is possible to observe the number of correctly classified audio samples for each activity and the number of wrongly classified samples. For the set of activities analyzed, the crying activity is the one that the model classifies in the best way, with 97.67% accuracy (166 of 170 samples correctly classified).

**Table 8:** Confusion matrix for the activity classification model.

	Cry	Play	Run	Walk
Cry	166	4	0	0
Play	7	195	2	1
Run	5	10	185	1
Walk	0	5	9	212

For the second model validation approach, the 10-fold cross-validation, Table 9 shows the accuracy and loss obtained for each fold and Table 10 shows the average scores for all folds. In both validation processes of the child activity classification model (70–30 division approach and 10-fold cross-validation) it can be observed that the accuracy is very similar.

**Table 9:** Scores per fold in the 10-fold cross-validation approach.

Fold	Accuracy (%)	Loss
1	92.91	0.8315
2	95.14	0.1704
3	92.50	1.1195
4	95.13	0.2525
5	97.00	0.1936
6	94.75	0.3846
7	94.00	0.2480
8	90.63	0.3185
9	93.63	0.6060
10	96.25	0.2308

**Table 10:** Average scores for all folds in the 10-fold cross-validation approach.

Accuracy (%)	Loss
94.19	0.4356

## Discussion

Although the substantial contribution of the present work is the presentation of a deep artificial neural network in the generation of a children activity classification model through environmental sound, there are aspects to be considered, such as the fact that the proposed architecture is specific for this model and is susceptible to being optimized.

The activities are independent of each other, that is, the analysis does not consider activities that are performed simultaneously. Composite activities would thus require a different analysis to be considered.

Another important point is the type of environmental sound with which one works, since this work is based on the fact that the captured audio corresponds to activities performed in environments with little or no external noise, and where only one individual (child) is performing an activity at the same time. Environments with considerable external noise or with several individuals (children) interacting at the same time would require another type of analysis.

It is also important to highlight that the results obtained in terms of accuracy for the two model validation approaches implemented in this work are similar, which confirms the performance of the model in the children activity classification using environmental sound.

## Conclusions

The aim of the present work was to create a children activity classification model using environmental sound as data source, through the design of a deep ANN, a well-known machine learning technique through which it is possible to generate activity recognition models, with significant accuracy. From the results shown in “Experiments and Results”, the following conclusions can be obtained:

- *Environmental sound can be used to correctly classify children activities*. Environmental sound is an effective data source for the generation of models for children activity classification, since it is possible to classify activities based on the analysis of the extracted features from the environmental sound.
- *Different-type activities are correctly classified*. The model correctly classifies activities of different types such as crying, playing, running and walking, unlike other models based on specific types of sensors (e.g., using accelerometers only for activities detectable by movement).
- *Deep artificial neural networks are efficient in generating children activity classification models through environmental sound*. The deep artificial neural network with the proposed architecture correctly classifies children activities with an accuracy of 94%, through the analysis of the extracted features from the environmental sound.
- *The accuracy of the deep artificial neural network is similar to other machine learning techniques reported*. The deep artificial neural network with the architecture proposed in the present work achieves an accuracy similar to that reported in our previous works, with other machine learning techniques: 100% for kNN with 34 features (Blanco-Murillo et al., 2018) and 94.25% for kNN with 27 features (Garca-Domnguez et al., 2019).

## Future Work

The present work allows us to demonstrate that a deep artificial neural network is an efficient technique in the generation of a children activity classification model, however, some aspects can be worked on in the future. Therefore, we propose as part of future work the analysis of the parameters described in the architecture of the neural network, as well as the consideration of feature selection techniques for the dataset. As for the set of activities analyzed, we also propose as future work the addition of more simple activities, as well as the proposal for the analysis of compound activities, both in controlled and uncontrolled environments. The proposed future work is:

- To analyze the parameters described in the architecture of the proposed deep artificial neural network with the aim of performing an optimization that allows increasing the accuracy in the classification of the activities.
- To include feature selection techniques to reduce the size of the dataset with which the deep artificial neural network works and this can favorably impact the performance of the model. This is important especially when working with models that are implemented in mobile devices with limited resources.
- To analyze the dataset size and the number of instances per class to ensure optimal training of the model and expand it if necessary.
- To increase the set of activities analyzed by adding additional activities that are performed by children, which will allow the model to be more robust.
- To propose the type of analysis to be performed in the case of compound activities.
- To analyze the methods and techniques to be used to classify children activities through environmental sound in uncontrolled environments with outside noise.
- To design a practical application on a specific scenario where the generated classification model can be applied considering the points to work mentioned above.

## Supplemental Information

10.7717/peerj-cs.308/supp-1 Supplemental Information 1 Deep neural network code and dataset. The code in python v2 of the implemented deep neural network and the dataset of the extracted features from the audio.

## Additional Information and Declarations

### Data Availability

The following information was supplied regarding data availability: Raw data are available in the Supplemental Files.
